# Regulation of SIRT3 signal related metabolic reprogramming in gastric cancer by *Helicobacter pylori* oncoprotein CagA

**DOI:** 10.18632/oncotarget.18695

**Published:** 2017-06-27

**Authors:** Do Yeon Lee, Dawoon E. Jung, Sung Sook Yu, Yeo Song Lee, Beom Ku Choi, Yong Chan Lee

**Affiliations:** ^1^ Department of Internal Medicine, Yonsei University College of Medicine, Seoul, Korea; ^2^ Institute of Gastroenterology, Yonsei University College of Medicine, Seoul, Korea; ^3^ Department of Biomedical Science, Yonsei University College of Medicine, Seoul, Korea; ^4^ Samsung Medical Research Center, Seoul, Korea; ^5^ Immune & Cell Therapy Branch, Division of Cancer Biology, National Cancer Center, Gyeonggi-do, Korea

**Keywords:** *helicobacter pylori*, CagA, SIRT3, HIF-1α, gastric cancer

## Abstract

Injection of the *Helicobacter pylori* cytotoxin-associated gene A (CagA) is closely associated with the development of chronic gastritis and gastric cancer. Individuals infected with *H. pylori* possessing the CagA protein produce more reactive oxygen species (ROS) and show an increased risk of developing gastric cancer. Sirtuins (SIRTs) are nicotinamide adenine dinucleotide (NAD^+^)-dependent deacetylases and mitochondrial SIRT3 is known to be a tumor suppressor via its ability to suppress ROS and hypoxia inducible factor 1α (HIF-1α). However, it is unclear whether increased ROS production by *H. pylori* is regulated by SIRT3 followed by HIF-1α regulation and whether intracellular CagA acts as a regulator thereof. In this study, we investigated correlations among SIRT3, ROS, and HIF-1α in *H. pylori*-infected gastric epithelial cells. We observed that SIRT3-deficient AGS cells induce HIF-1α protein stabilization and augmented transcriptional activity under hypoxic conditions. In CagA^*+*^ *H. pylori* infected cells, CagA protein localized to mitochondria where it subsequently suppressed SIRT3 proteins. CagA^*+*^ *H. pylori* infection also increased HIF-1α activity through the ROS production induced by the downregulated SIRT3 activity, which is similar to the hypoxic condition in gastric epithelial cells. In contrast, overexpression of SIRT3 inhibited the HIF-1α protein stabilization and attenuated the increase in HIF-1α transcriptional activity under hypoxic conditions. Moreover, CagA^*+*^ *H. pylori* attenuated HIF-1α stability and decreased transcriptional activity in SIRT3-overexpressing gastric epithelial cells. Taken together, these findings provide valuable insights into the potential role of SIRT3 in CagA^*+*^ *H. pylori*-mediated gastric carcinogenesis and a possible target for cancer prevention via inhibition of HIF-1α.

## INTRODUCTION

Previous studies have demonstrated that hypoxia is an important microenvironmental factor in promoting tumor progression [1]. Gastric cancers are exposed to hypoxia, as are many other solid tumors. Epidemiological studies have suggested that the development of gastric cancer may be attributed to hypoxia-induced reactive oxygen species (ROS) [2] and that ROS generated within the gastric mucosa are related to continuous exposure to *H. pylori* infection, ingested food, and cigarette smoking, etc. Accumulating data indicate that the *H. pylori* CagA protein, which is injected into gastric epithelial cells through T4SS, behaves as a bacterial oncoprotein [3]: CagA continuously dysregulates multiple oncogenic signaling pathways and promotes tumorigenesis [4]. Suzuki *et al.* found that ROS production in gastric epithelial cells was significantly enhanced by infection with CagA-positive *H. pylori* strains, resulting in an extensive accumulation of neutrophils [5], and was involved in tumor initiation, enhanced expression of oncogenes, and increased cell proliferation.

Increased ROS production may be involved in a variety of cellular changes, including changes in metabolism. Alterations in metabolism can help cancer cells survive various stresses, such as hypoxia and a limited supply of glucose. Some of the metabolic changes are facilitated by the transcription factor hypoxia inducible factor 1α (HIF-1α) [6]. HIF-1α activation is dependent on oxygen levels. Under normoxia, HIF-1α is hydroxylated on proline residues by prolyl hydroxylase domain proteins (PHDs) and degraded by proteasomes. Under hypoxia, HIF-1α is stabilized and translocated into the nucleus where it binds to the hypoxia-response element (HRE) in the promoters of target genes [1, 7]. Mitochondrial electron transport chain-generated ROS can also stabilize HIF-1α, resulting in the transcription of genes involved in glucose transport and glycolytic enzymes, as well as promoting cell proliferation [8, 9].

Several members of the sirtuin family (SIRT1-7), the human homologues of the *Sir2* gene in yeast, have been reported to play important roles in carcinogenesis [10]. Sirtuins are a family of nicotinamide adenine dinucleotide (NAD^+^)-dependent protein deacetylases [11]. Sirtuins regulate multiple cellular processes and physiological states, including oxidative stress, genomic stability, cell survival, development, metabolism, aging, and longevity [12, 13]. Of the seven SIRT analogues, SIRT3, SIRT4, and SIRT5 are localized in the mitochondria [14]. Strikingly, SIRT3 deacetylates and activates several enzymes involved in cellular redox balance and defense against oxidative damage [15–18]. In addition, SIRT3 knock-out (KO) murine embryonic fibroblasts (MEFs) have been found to cause a shift towards glycolytic metabolism, exhibiting faster glucose uptake, lower levels of TCA intermediates, higher levels of lactate, and significantly faster proliferation, compared to wild-type MEFs [19, 20]. Recently, SIRT3 was reported to act as a mitochondrial localized tumor suppressor via its ability to inhibit mitochondrial ROS production. Loss of SIRT3 has been found to increase the production of ROS and to lead to HIF-1α stabilization under hypoxic conditions. In contrast, SIRT3 overexpression has been shown to impede HIF-1α stabilization in hypoxia and to inhibit tumorigenesis [19, 21, 22]. To our knowledge, the role of SIRT3 in *H. pylori*-induced gastric carcinogenesis has not been investigated. In this study, we sought to investigate whether SIRT3 plays a role in the ROS production induced by the *H. pylori* oncoprotein CagA and whether increased ROS can affect HIF-1α activation leading to *H. pylori*-mediated gastric carcinogenesis.

Herein, we show that loss of SIRT3 in gastric epithelial cells increases HIF-1α protein stabilization and its transcriptional activity. Infection with *H. pylori* CagA induced downregulation of SIRT3 protein in mitochondria, stimulated ROS production, and elicited HIF-1α stabilization with increased transcriptional activity, similar to that observed during hypoxia. Meanwhile, however, SIRT3-overexpressing gastric epithelial cells inhibited the stabilization of HIF-1α protein in hypoxia and attenuated the observed increases in HIF-1α transcriptional activity in hypoxia. Moreover, *H. pylori* CagA attenuated HIF-1α stability and its transcriptional activity in SIRT3-overexpressing gastric epithelial cells. These findings suggest that *H. pylori* CagA induces HIF-1α activity by downregulating SIRT3, followed by increases in ROS production, which provides a novel mechanism to explain the pathogenesis of *H. pylori*-mediated gastric carcinogenesis.

## RESULTS

### SIRT3 knockdown increases proliferation of gastric epithelial cells and HIF-1α activity through ROS generation

**Figure 1:**
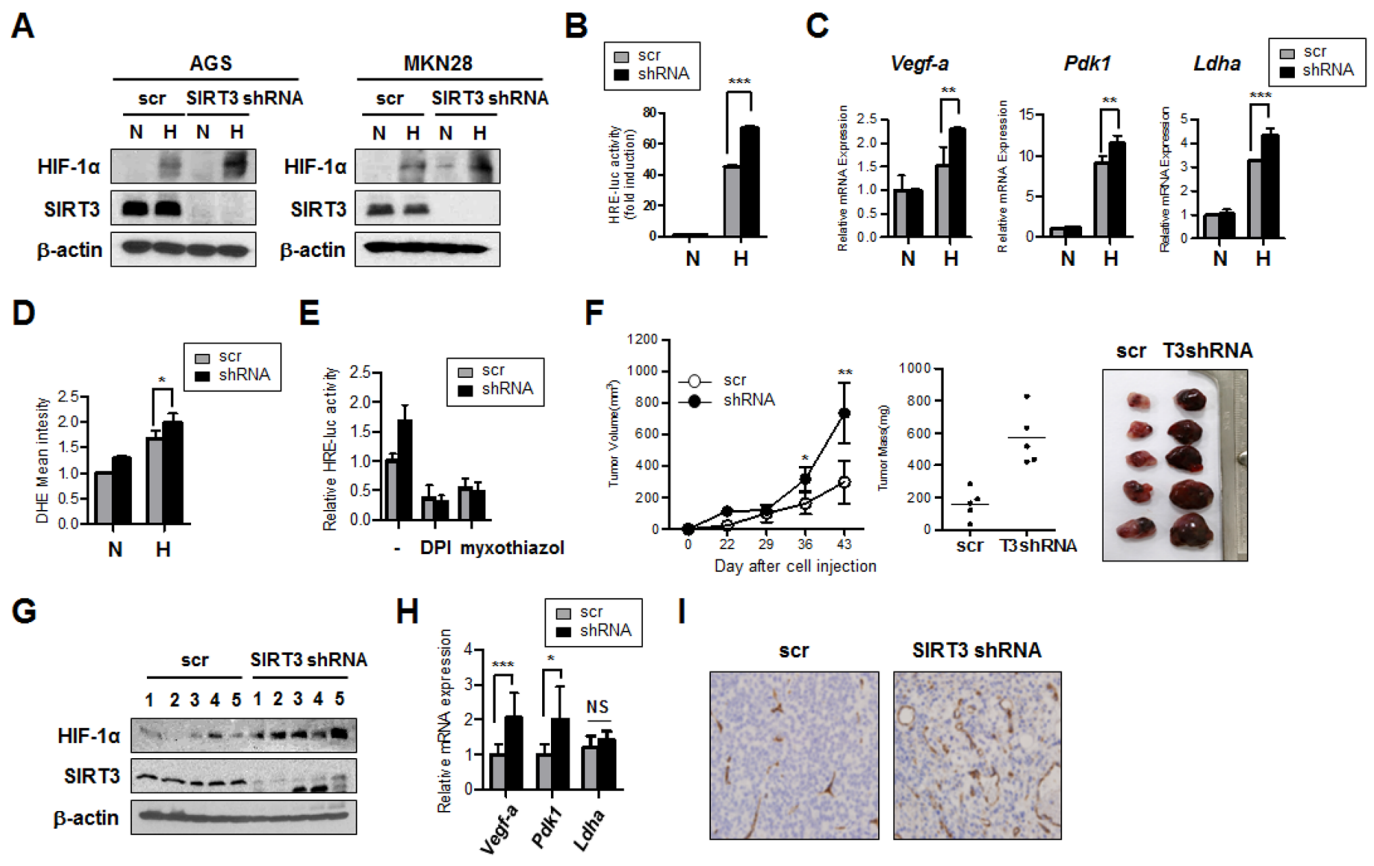
SIRT3 knockdown in gastric epithelial cells increases HIF-1α activity and induces tumor growth **(A)** SIRT3 and HIF-1α protein levels were detected by immunoblotting extracts from AGS and MKN28 cells stably expressing a scrambled control shRNA (scr) or SIRT3 shRNA at normoxic (N, 21% O_2_) or hypoxic (H, 1% O_2_) conditions for 12 h. **(B)** SIRT3 stable knockdown cell lines (shRNA) or a scrambled control vector (scr) line were transfected with HRE-luciferase for 24 h and incubated in normoxic or hypoxic conditions for 16 h, after which luciferase reporter activity was measured. **(C)** RNA was isolated from AGS cells stably expressing SIRT3 shRNA or a scrambled control vector (scr) cultured in normoxic or hypoxic conditions for 16 h. The expression of HIF-1α target genes in response to hypoxia was measured by real-time PCR using specific primers for *Vegf-a*, *Pdk-1,* and *Ldha.* **(D)** ROS levels were measured by FACS analysis for AGS cells with SIRT3 knockdown or a scrambled control (scr) cultured in normoxic or hypoxic conditions for 12 h. **(E)** Scrambled or SIRT3 shRNA stable cell lines were transfected with HRE-luciferase for 24 h, treated with inhibitors DPI or myxothiazol for 1 h, and then, incubated in normoxic or hypoxic conditions for 16 h. Firefly luciferase reporter activity was measured and normalized to Renilla luciferase activity. **(F)** AGS cells expressing control or SIRT3 shRNA were subcutaneously injected into the flanks of nude mice (n=5). Xenograft tumor volumes were monitored weekly and growth curves were plotted (left panel). Xenograft tumors were excised and weighed at the end of the experiment (middle panel). Pictures of dissected tumors are shown in the right panel. Each bar indicates the mean ± S.E. (n=5; *p<0.05; **p<0.01; *** p < 0.001). **(G)** Tumor samples from nude mice injected with control, and SIRT3 shRNA AGS cells were lysed and immunoblotted with anti-HIF-1α and SIRT3. **(H)** *Vegf-a* and *Pdk1* expression levels were measured by real-time PCR with isolated total RNA from tumor samples. **(I)** Tumor samples were collected, and immunohistochemical staining was performed with anti-CD31 antibody. Data are represented as mean ± SD (n = 3). *p<0.05; **p<0.01; *** p < 0.001.

Loss of SIRT3 has been shown to increase mitochondrial ROS production and HIF-1α activity under hypoxic conditions [22]. We sought to investigate whether SIRT3 knockdown plays a role in the progression of gastric cancer using established gastric epithelial cell lines. We knocked down SIRT3 in the gastric epithelial cells with short-hairpin (sh)RNA and explored whether SIRT3 knockdown affected the hypoxic activation of HIF-1α. We found that SIRT3 knockdown in gastric epithelial cells under hypoxic conditions resulted in increased expression of HIF-1α protein, compared to the scrambled control (Figure 1A). We next examined whether there is a concomitant increase in HIF-1α transcriptional activity using the reporter construct with hypoxia responsive element (HRE) [22]. Under hypoxic conditions, there was a 40-fold increase in HRE-luciferase activity in the scrambled control, compared with normoxic conditions, whereas the SIRT3 knockdown cells demonstrated a 70-fold increase in luciferase activity under the same conditions (Figure 1B). Consistent with this, the expression of HIF-1α target genes, such as *Vegf-a*, *Pdk1*, and *Ldha*, were significantly elevated, compared to the control, during hypoxia, and the loss of SIRT3 further increased expression of these HIF-1α target genes (Figure 1C). Hypoxic conditions have been reported to increase ROS production, and these higher ROS levels are sufficient to stabilize and activate HIF-1α [22, 23]. As expected, we also observed increased ROS levels in SIRT3 knockdown AGS cells, compared with the control, in hypoxic conditions (Figure 1D). To determine whether the source of the increased ROS production in the absence of SIRT3 was complex I or III of mitochondria, we measured HIF-1α activity by luciferase assay in the presence or absence of selective inhibitors of mitochondrial complex I or complex III. DPI has been used to inhibit ROS production mediated by plasma membrane NAD(P)H oxidase [24, 25] and has also been reported to inhibit the production of superoxide and H_2_O_2_ by mitochondria through inhibiting NADH-ubiquinone oxidoreductase (complex I) [26, 27]. Myxothiazol binds at the quinol oxidation (Qo) site of the bc1 complex (complex III) to block electron transfer, and impairs ROS production [28]. We found that addition of inhibitors attenuated the increase in HRE-luciferase activity in the SIRT3 knockdown cells cultured under hypoxic conditions (Figure 1E), thereby demonstrating that the increase in HIF-1α activity with hypoxic conditions is due to ROS generated by the mitochondrial electron transport chain. To demonstrate the role of SIRT3 in the progression of tumors, we injected control and SIRT3 knockdown AGS cells into the flanks of nude mice, and tumor growth was measured after the subcutaneous injection. We found that tumor volume derived from SIRT3 knockdown cells was significantly increased, compared with control cells (Figure 1F). In addition, HIF-1α protein levels and the expression of HIF-1α target genes were analyzed, and the expression of *Vefg-a* and *Pdk1* were significantly increased in SIRT3-deficient tumor tissues, compared with the controls, as was the degree of angiogenic activity, as determined by immunostaining for the endothelial cell-specific marker CD31 (Figure 1G-1I). Taken together, these results indicate that SIRT3 loss is linked to tumorigenesis mediated via ROS-induced HIF-1α activity, leading to enhanced angiogenesis and glycolytic pathway metabolism.

### Infection with *H. pylori* CagA increases HIF-1α activity and expression of HIF-1α target genes

**Figure 2:**
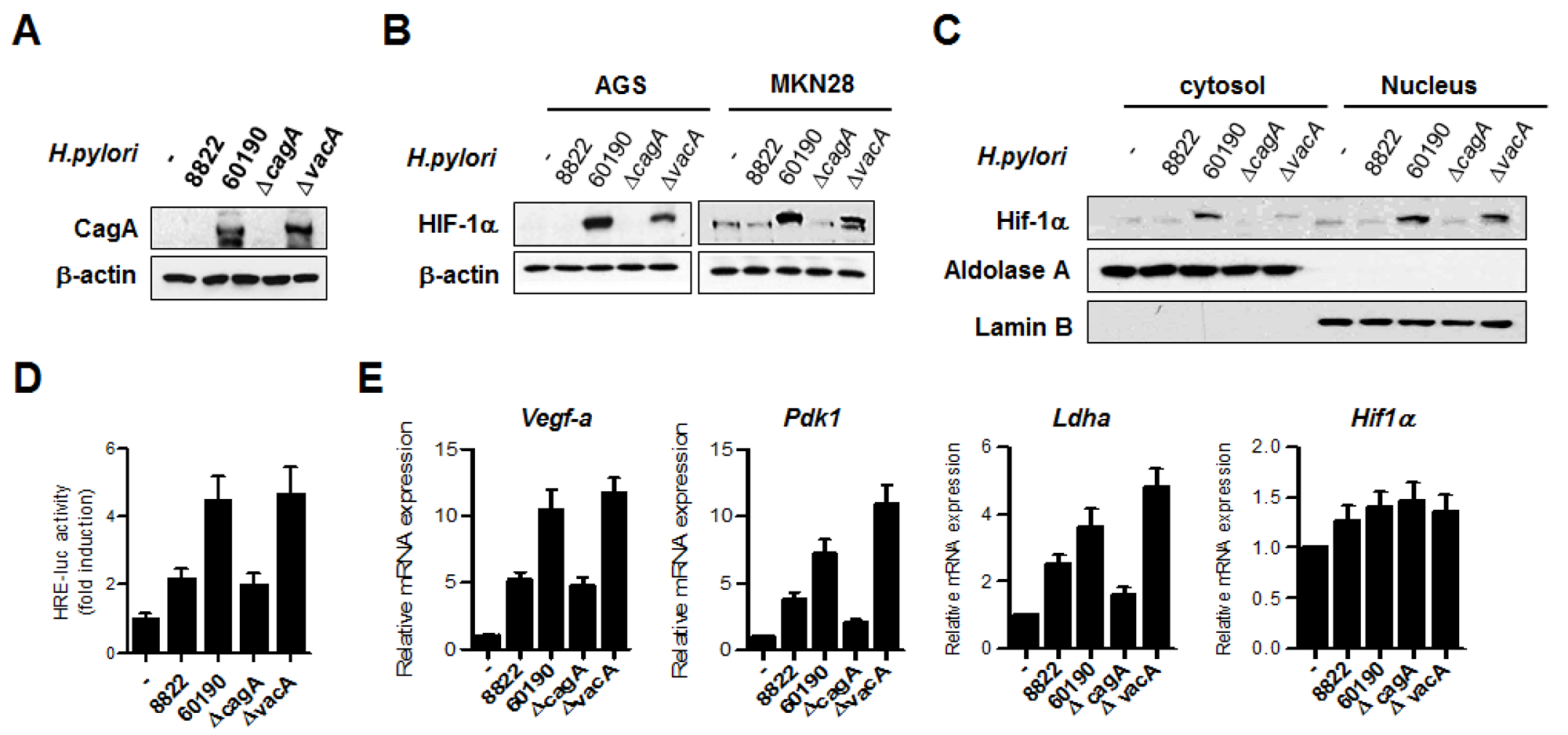
*H. pylori* CagA increases HIF-1α activity and induces expression of HIF-1α target genes **(A)** AGS cells were infected with *H. pylori* strains for 6 h and the CagA protein level was analyzed by immunoblotting. **(B)** Lysates from AGS and MKN28 cells infected with *H. pylori* strains were immunoblotted with anti-HIF-1α, and then, the membrane was stripped and analyzed with anti-β-actin to standardize gel loading. **(C)** MKN28 cells were infected with *H. pylori* strains for 6 h, and then, cytoplasmic and nuclear fractions were separated. The nuclear proteins were analyzed by immunoblotting with anti-HIF-1α. To verify complete separation of the cytosolic and nuclear fractions, cytosolic and nuclear extracts were immunoblotted for aldolase A and lamin B. **(D)** MKN28 cells were co-transfected with HRE-Luc together with TK-renilla for 24 h and then infected with *H. pylori* strains for 12 h. Firefly luciferase activity was measured and normalized to Renilla luciferase activity. **(E)** MKN28 cells were infected with *H. pylori* for 12 h, and the fold change in HIF-1α target gene levels was measured by real-time PCR using specific primers for *Vegf-a*, *Pdk-1, Ldha,* and *Hif-1α*. Data are represented as mean ± SD (n = 3).

To examine HIF-1α protein expression in relation to the oncoprotein CagA secreted by *H. pylori*, extracts from cells infected with *H. pylori* strains were immunoblotted using HIF-1α antibody. In this experiment, we used four strains of *H. pylori* (two CagA-negative and two CagA-positive strains) and confirmed the expression of CagA protein in CagA^+^ strains (60190 and Δ*vacA* 60190) (Figure 2A). Surprisingly, stronger expression of HIF-1α protein was observed in CagA^+^ *H. pylori* strains than in the CagA^-^ strains (Figure 2B, Supplementary Figure 1). Immunoblotting of cytosolic and nuclear fractions showed that infection with CagA^+^ *H. pylori* induced greater HIF-1α nuclear translocation than CagA^-^ *H. pylori* (Figure 2C). In line with this finding, the trancriptional activity of HIF-1α was increased on infection with CagA^+^ *H. pylori* strains in accordance with *H. pylori* CagA expression status (Figure 2D). In addition, expression levels of the HIF-1α target genes *Vegf-a*, *Pdk1,* and *Ldha* were higher in CagA^+^ *H. pylori*, compared to CagA^-^ *H. pylori*, without affecting *Hif-1α* mRNA levels (Figure 2E). These results suggest that infection with CagA^+^ *H. pylori* stimulates HIF-1α in gastric cells similar to that observed in the hypoxic response.

### *H. pylori* CagA infection downregulates SIRT3 activity in mitochondria via proteasomal degradation

**Figure 3:**
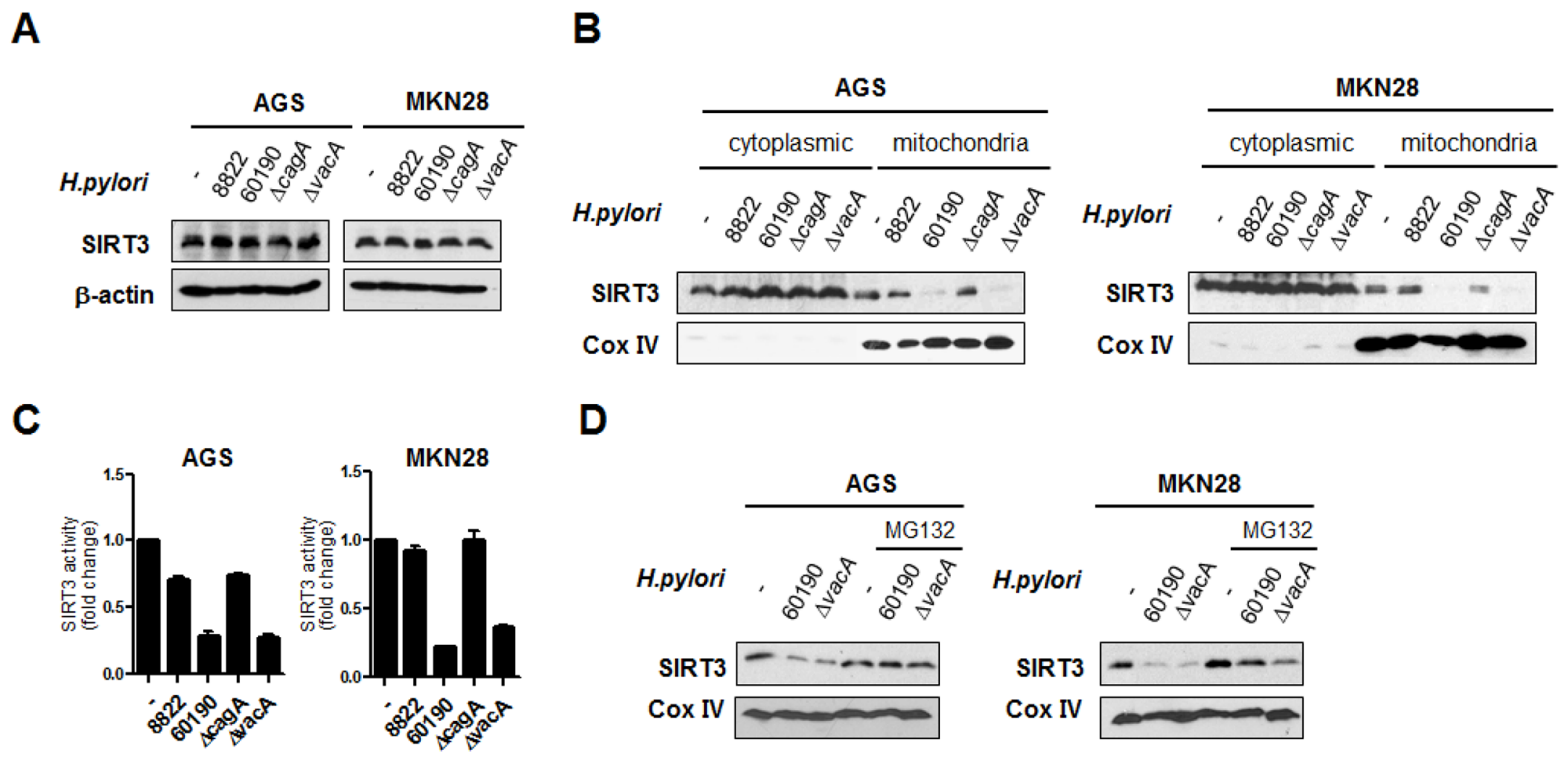
*H. pylori* CagA downregulates SIRT3 activity in the mitochondria via proteasomal degradation **(A)** SIRT3 protein levels were detected by immunoblotting from AGS and MKN28 cells infected with *H. pylori* for 6 h. **(B)** Cytoplasmic (Cyt) and mitochondrial (Mito) proteins were fractionated from *H. pylori*-infected AGS and MKN28 cells after 6 h and analyzed by immunoblotting. Cox IV, a known mitochodrial protein, was detected as a control. **(C)** Relative activity of SIRT3 was measured using a fluorimetric method from the mitochondrial extracts of AGS and MKN28 cells infected with *H. pylori* for 6 h. Values represent fold changes relative to the control. **(D)** AGS or MKN28 cells were pretreated with MG132 (10 μM) for 1 h prior to infection with CagA^+^ *H. pylori* strains. The mitochondrial fraction was subjected to immunoblot analysis using anti-SIRT3 antibody. As a loding control, blots were stripped and reprobed with anti-Cox IV. Data are represented as mean ± SD (n = 3).

To determine whether there were any changes in SIRT3 expression following infection with various *H. pylori* strains, we examined whole cell SIRT3 protein levels in *H. pylori*-infected cells. Total SIRT3 protein levels were not altered in *H. pylori*-infected cells (Figure 3A). We decided to measure SIRT3 protein levels and their enzymatic activity in the mitochondria by preparing mitochondrial extracts from *H. pylori*-infected cells. Interestingly, the protein levels and enzymatic activity of SIRT3 in mitochondria were significantly reduced upon infection with CagA^+^ *H. pylori*, compared with CagA^-^ *H. pylori* (Figure 3B and 3C). The low level of SIRT3 protein in mitochondria of CagA^+^ *H. pylori*-infected cells was intriguing. Some proteins involved in bioenergetic metabolism localized in the mitochondria (p53, p70, ODC, SOD1, etc.) are known to be degraded by an ubiquitin-independent process in response to oxidative stress [29, 30]. We investigated whether proteasomal degradation could contribute to the low level of mitochondrial SIRT3 protein in CagA^+^ *H. pylori*-infected cells. We pretreated cells with the specific proteasome inhibitor MG132 prior to *H. pylori* infection and found that MG132 was able to block SIRT3 protein degradation in cells (Figure 3D).

### Increased ROS production induced by *H. pylori* CagA infection regulates HIF-1α stability

**Figure 4:**
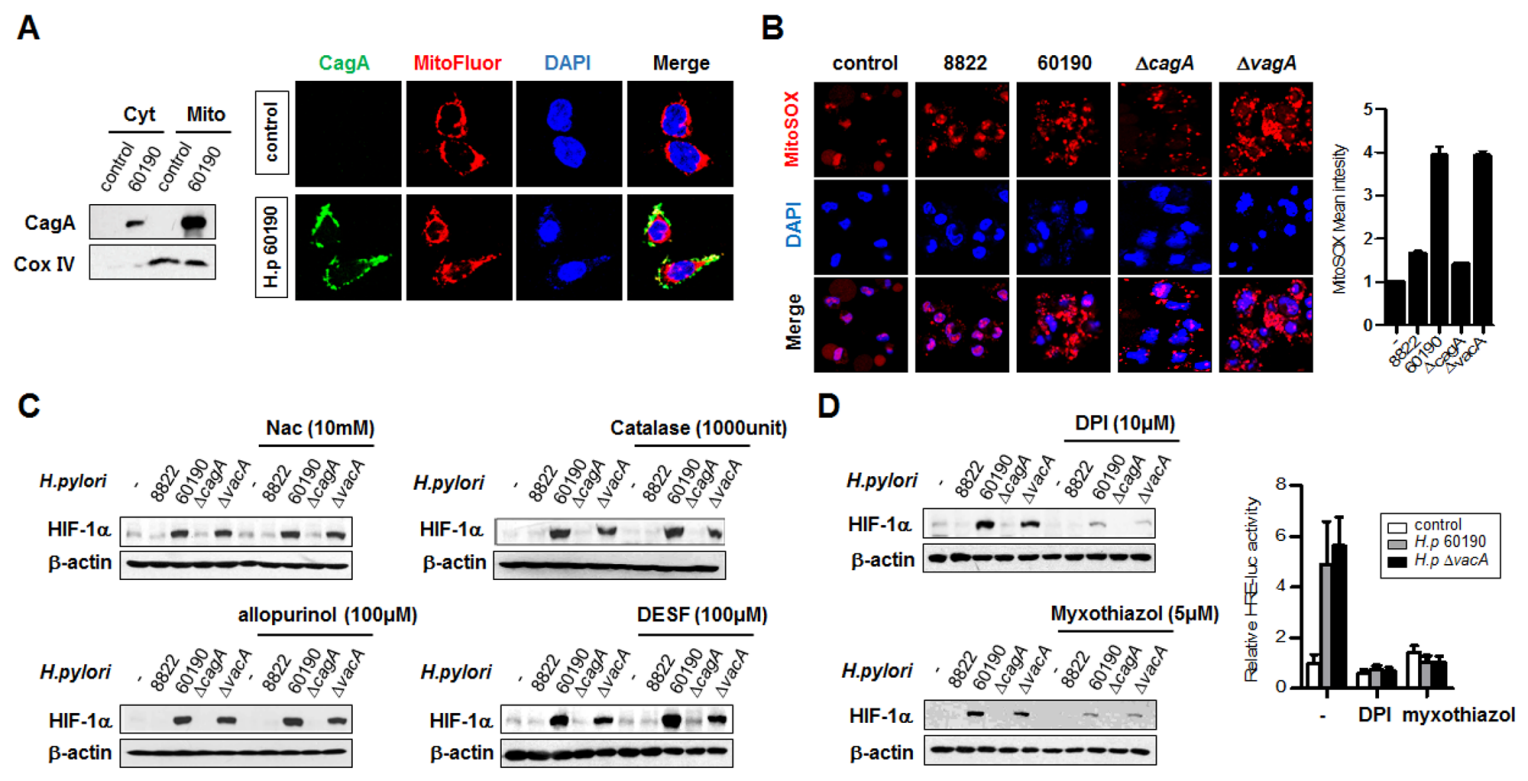
*H. pylori* CagA protein localizes to mitochondria and is involved in ROS production **(A)** Cytoplasmic (Cyt) and mitochondrial (Mito) proteins were fractionated from *H. pylori*-infected AGS cells and analyzed by immunoblotting (left panel) and immunofluorescence (right panel) with anti-CagA antibody. In immunofluorescence microscopy, CagA was stained in green, mitochondria in red, and DNA in blue with 4’,6-diamidino-2-phenylindole (magnification x100). **(B)** AGS cells were infected with *H. pylori* for 6 h and stained with Mitosox, followed by immunofluorescence (left panel) and FACS analysis (right panel). **(C)** AGS cells were pretreated with antioxidants (NAC, catalase, allopurinol, and DESF) prior to *H. pylori* infection and extracts were immunoblotted with HIF-1α antibody. **(D)** AGS cells were pretreated with DPI or myxothiazol and then the extracts were immunoblotted (left panel). AGS cell were co-transfected with HRE-Luc and TK-renilla constructs for 24 h and treated with the inhibitors DPI or myxothiazol for 1 h. Cells were then infected with CagA^+^ *H. pylori* strains for 6 h, and luciferase reporter activity was measured. Data are represented as mean ± SD (n = 3).

A fraction of expressed CagA protein has been reported to localize to mitochondria and produce significant amounts of ROS in infected cells. The mechanism by which CagA induces ROS production in gastric epithelial cells is not known [2, 31]. We thus examined whether *H. pylori* CagA protein localizes to mitochondria and increases ROS production. Immunoblotting after subcellular fractionation revealed CagA protein in both cytoplasmic and mitochondrial fractions (Figure 4A, left panel). In immunofluorescence analysis, with the CagA protein stained green and mitochondria stained red, yellow showed the colocalization of CagA and mitochondria in cells, consistent with the immunoblotting findings (Figure 4A, right panel). Next, we measured ROS levels using immunofluorescence assays and FACS analysis in AGS cells infected with the relevant *H. pylori* strains. Interestingly, we observed an increase of ROS in CagA^+^ *H. pylori* strains (Figure 4B), suggesting that CagA protein localized to the mitochondria has the potential to produce ROS. To determine whether the activation of HIF-1α is due to ROS, we treated AGS cells with antioxidants and determined the extent of HIF-1α activation. GSH precursor N-acetyl-cysteine (NAC), catalase, allopurinol, and DESF were added to cells before *H. pylori* infection. These antioxidants failed to downregulate HIF-1α expression (Figure 4C), implying that these antioxidants did not effectively scavenge the secreted extracellular oxidant in these cells. In order to determine the cellular source of ROS, DPI (complex I inhibitor) and myxothiazol (complex III inhibitor) were used to pretreat the cells before infection with *H. pylori*. Both inhibitors downregulated the stability and transcriptional activity of HIF-1α, indicating that the increased HIF-1α activity in CagA^+^ *H. pylori*-infected AGS cells was mainly due to ROS produced by mitochondrial complex I and III (Figure 4D).

### CagA^+^ *H. pylori-*induced HIF-1α stabilization is regulated by post-translational modification

**Figure 5:**
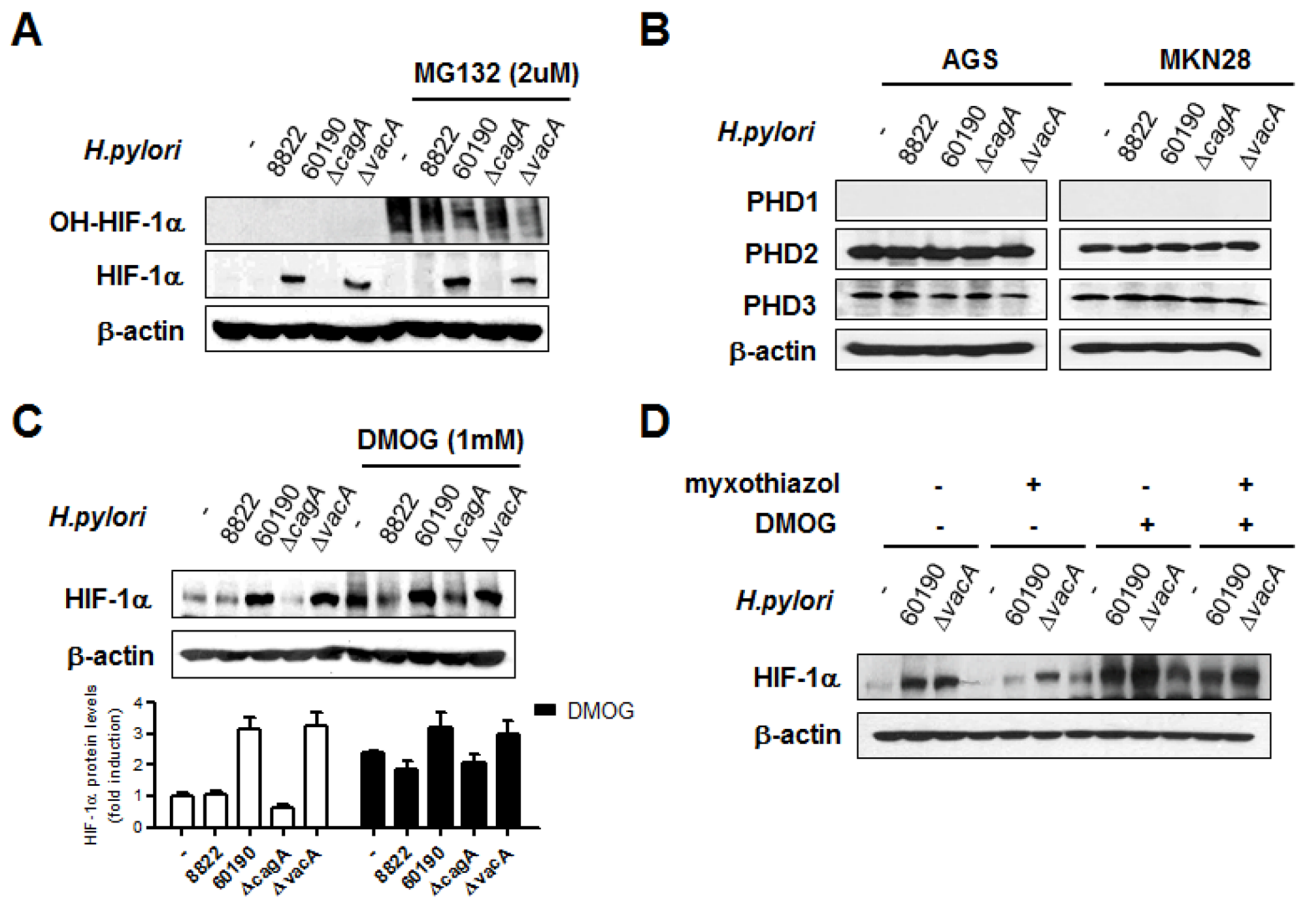
CagA^+^ *H. pylori*-induced HIF-1α stabilization is regulated by prolyl hydroxylase (PHD) activity **(A)** AGS cells were pretreated with or without 10 μM MG-132 for 1 h and infected with *H. pylori* for 6 h. Cell lysates were immunoblotted with antibodies specific for hydroxylated HIF-1α (OH-HIF-1α) and total HIF-1α. **(B)** After infection with *H. pylori* for 6 h, immunoblotting was performed using the indicated antibodies. **(C)** AGS cells were pretreated with or without 1mM DMOG for 2 h and infected with *H. pylori* for 6 h. Cell lysates were immunoblotted with anti-HIF-1α antibody. Densitometric ratios between HIF-1α and β-actin immunoblotting are shown at the bottom. **(D)** After treatment with the indicated inhibitors prior to CagA^+^ *H. pylori* infection, HIF-1α protein levels were detected by immunoblotting. Data are represented as mean ± SD (n = 3).

We next tested the hypothesis that CagA-induced HIF-1α stabilization may be due to post-translational modification, because there were no significant differences in HIF-1α mRNA levels in *H. pylori*-infected cells. We assessed HIF-1α stability by treating cells with MG-132 to prevent hydroxylated HIF-1α from being degraded. Although CagA^+^ *H. pylori* infection stabilized more HIF-1α during MG-132 treatment, cells had significantly less hydroxylated HIF-1α, indicating that PHD activity was lower in CagA^+^ *H. pylori*-infected cells, compared to CagA^-^ cells (Figure 5A). Of all the PHDs, PHD2 and PHD3 were detected in both gastric epithelial cell lines (Figure 5B), and PHD2 is known to be the primary mediator of hydroxylation [32]. Since CagA^+^ *H. pylori* infection influences HIF-1α stability through modulation of PHD activity, pretreatment with the potent PHD inhibitor DMOG in CagA^-^ *H. pylori-*infected cells, as expected, showed relatively restored HIF-1α stabilization (Figure 5C). These results suggested that increased HIF-1α expression contributes to reduced PHD activity in CagA^+^ *H. pylori*-infected cells (Figure 5D). CagA^+^ *H. pylori* activates HIF-1α through ROS-mediated alteration of PHD function, thereby altering hydroxylation and subsequent proteasomal degradation of HIF-1α.

### SIRT3 gain of function inhibits HIF-1α activity and expression of HIF-1α target genes

**Figure 6:**
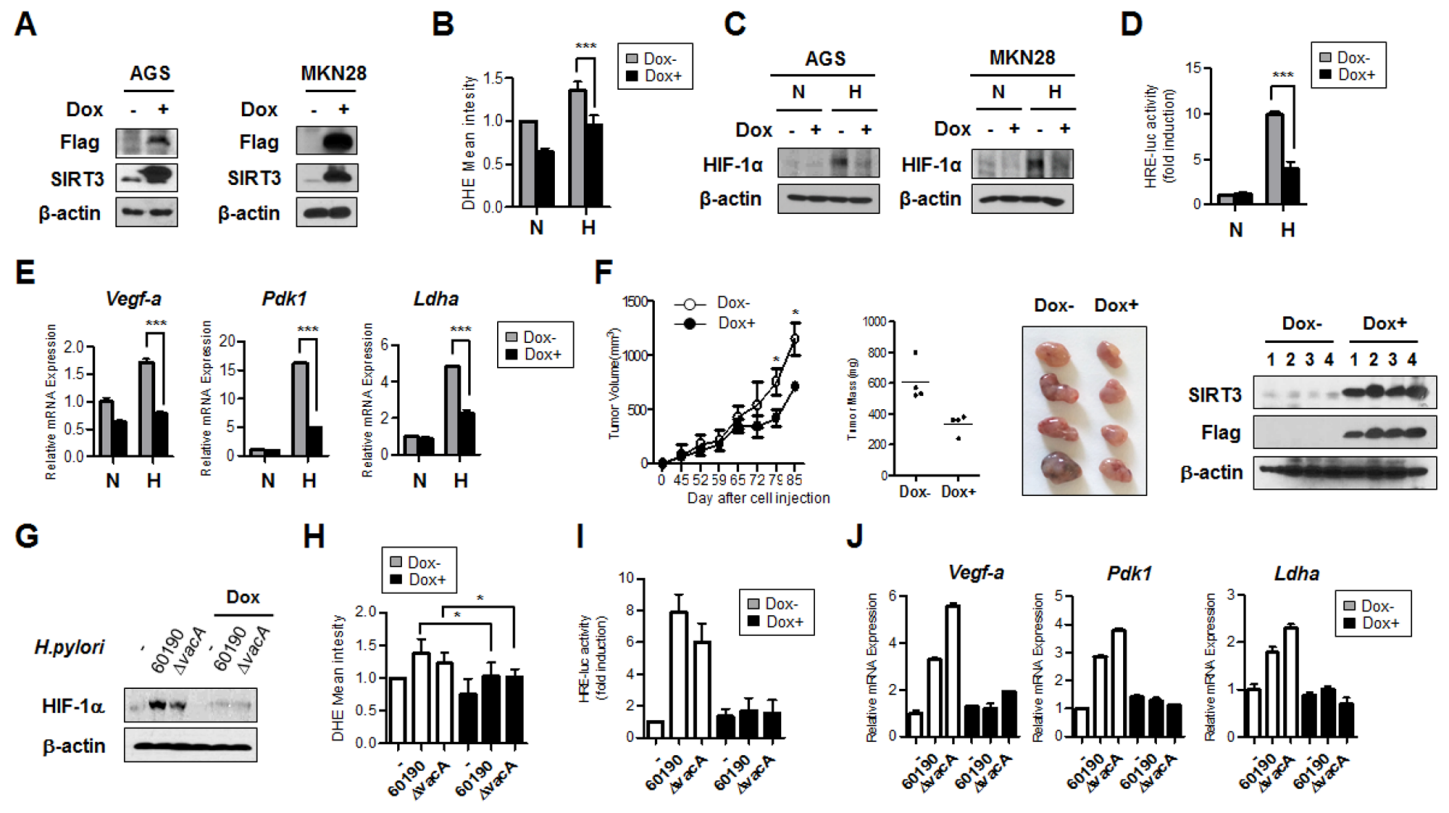
SIRT3 gain of function inhibits HIF-1α activity and expression of HIF-1α target genes **(A)** After generating derivatives of the gastric cancer cells AGS and MKN28 stably expressing doxycycline-inducible SIRT3, doxycycline (2 μM) was added to the cells for 48 h. Immunoblotting was then performed to detect SIRT3 overexpression using anti-Flag antibody. **(B)** Cells with inducible overexpression of SIRT3 were incubated in the presence or absence of Dox under normoxic (N, 21% O_2_) or hypoxic (H, 1% O_2_) conditions for 12 h, and then FACS analysis was performed to measure the amount of ROS. **(C)** For the immunoblotting assay, cells with inducible overexpression of SIRT3 in the presence or absence of Dox were incubated in normoxic or hypoxic conditions for 12 h. For the luciferase reporter assay **(D)** and for real time PCR **(E)** similar cells were incubated in normoxic or hypoxic conditions for 16 h. **(F)** Tumor volume and mass (left two panels) of SIRT3-overexpressing cells were measured. Pictures of tumors (third panel) are shown, and confirmation of SIRT3 overexpression by immunoblotting using lysates of the tumor samples (fourth panel). Each bar indicates the mean ± S.E. (n=4; *p<0.05). **(G)** After overexpressing SIRT3 in AGS cells with dox for 48 h, cells were infected with CagA^+^ *H. pylori* strains for 6 h. The HIF-1α protein level was detected by immunoblot analysis. **(H)** ROS levels were measured by FACS analysis. **(I)** HIF-1α transcriptional activity was measured by luciferase assay. **(J)** The expression levels of HIF-1α target genes (*Vegf-1a*, *Pdk1*, and *Ldha*) were measured by real-time PCR. Data are represented as mean ± SD (n = 3). * p< 0.05, ** p< 0.01, *** p< 0.001.

We generated AGS and MKN28 cells stably expressing doxycycline-inducible SIRT3 and confirmed SIRT3 overexpression after the addition of doxycycline by immunoblotting (Figure 6A). By FACS analysis, SIRT3-overexpressing cells showed decreased ROS levels, compared to control cells, in the hypoxic condition (Figure 6B). When induced by doxycycline treatment, cells overexpressing SIRT3 clearly had both reduced HIF-1α protein stability and HIF-1α transcriptional activity under hypoxia, compared with the control (Figure 6C and 6D). Expression of the HIF-1α responsive target genes *Vegf-a*, *Pdk1,* and *Ldha* was attenuated, corresponding to reduced HIF-1α activity, in SIRT3-overexpressing cells (Figure 6E). To examine whether SIRT3 has a role in inhibiting tumor growth *in vivo*, we injected SIRT3-overexpressing AGS cells into the flanks of nude mice. Tumor weights and volumes derived from SIRT3-overexpressing cells were smaller than those from the un-induced cells (Figure 6F).

**Figure 7:**
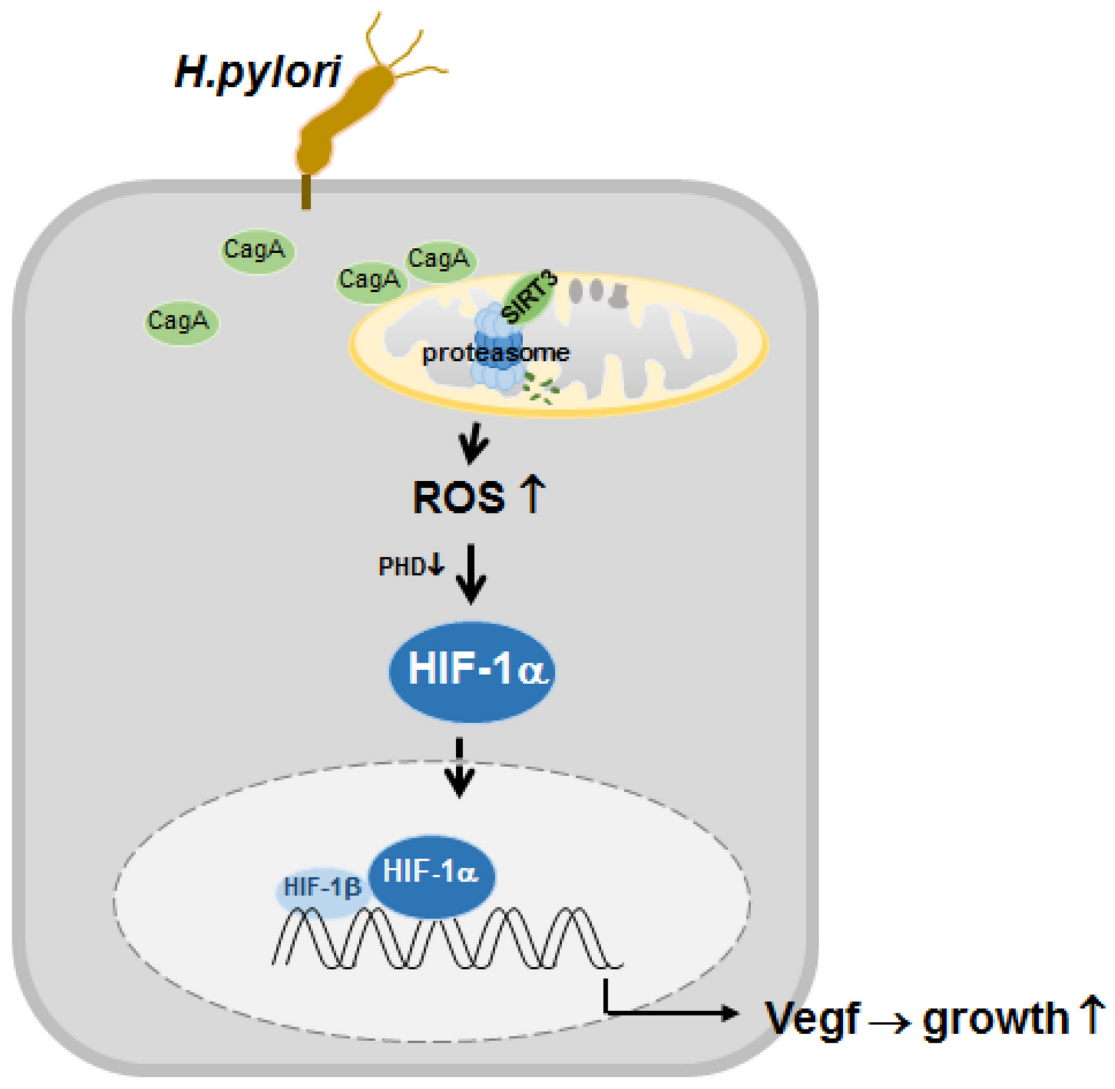
Proposed model depicting the regulation of HIF-1α by downregulated SIRT3-mediated ROS production in *H. pylori*-infected gastric cells Infection with *H. pylori* CagA^+^ induces SIRT3 protein degradation by proteasome activity. Increased ROS generation from the downregulated SIRT3 activity mediates the activation of HIF-1α, and this, in turn, induces the expression of HIF-1α target genes, which leads to gastric cancer proliferation.

CagA^+^ *H. pylori* infection showed increased HIF-1α stabilization and enhanced activity. We sought to determine the effect of SIRT3 overexpression on CagA^+^ *H. pylori*-induced HIF-1α stabilization. We found that the level of HIF-1α protein was reduced in SIRT3-overexpressing cells infected with CagA^+^ *H. pylori* (Figure 6G). ROS levels, HIF-1α transcriptional activity, and expression of HIF-1α target genes were also downregulated in CagA^+^ *H. pylori*-infected SIRT3-overexpressing cells (Figure 6H-6J), indicating that SIRT3 is an effector molecule responsible for negative regulation of ROS, HIF-1α, and tumor growth induced by *H. pylori* CagA. All of these findings suggest a novel mechanism whereby downregulation of SIRT3 in *H. pylori* CagA^+^ infected gastric cells induces ROS, HIF-1α activity, and tumor growth (Figure 7).

## DISCUSSION

This study was designed to answer the question whether the tumor suppressor SIRT3 protein influences ROS production and HIF-1α activity in *H. pylori*-infected gastric cancer cells. Using the established SIRT3 knockdown or SIRT3 overexpressing gastric epithelial cells to investigate the role of SIRT3, we confirmed results from previous studies that suggested that SIRT3 functions as a tumor suppressor due to the suppression of ROS-mediated HIF-1α activation [22].

Next, we examined the role of SIRT3 in regulating ROS/HIF-1α in *H. pylori*-infected gastric cells *in vitro*. In this study, *H. pylori* infection showed higher ROS production and HIF-1α activation in an oncoprotein CagA-dependent manner. The increased ROS triggered by CagA^+^ *H. pylori* might be attributed to the localization of CagA to the mitochondria (Figure 4A and 4B), with subsequent deregulation of the mitochondrial electron transport chain and production of superoxide [2, 31]. Vacuolating cytotoxin (VacA) protein, another known virulence factor of *H. pylori*, via pinocytosis and intracellular trafficking, induces vacuolation and epithelial cell apoptosis and this effect has been attributed to interactions with mitochondria [33–34]. It has been understood that VacA is capable of inducing an influx of Ca^2+^, resulting in the leakage of mitochondrial cytochrome c and the generation of reactive oxygen intermediates (ROI) [35–41]. We, however, observed that CagA induced significantly greater ROS production than VacA. This suggests that ROS-induced HIF-1 activation in *H. pylori* infected gastric cell lines is influenced by CagA protein more than VacA.

Interestingly, we found that antioxidants, such as NAC, catalase, allopurinol, etc., were unable to efficiently scavenge the secreted ROS in CagA^+^ *H. pylori*-infected gastric cells. Other reports have demonstrated that the amounts of catalase and superoxide dismutase (SOD) released by *H. pylori* are likely insufficient to clear excess extracellular oxidants [42] and that regulating the source of ROS generation is likely to be a more effective means of reducing ROS production. Therefore, we propose that impairment in the mitochondrial electron transport complex I or III by *H. pylori* CagA translocation results in increased ROS production and gives rise to a positive loop between ROS and HIF-1α.

It was previously reported that HIF-1α has a carcinogenic role in gastric cancer (43-44). Griffiths *et al.* investigated HIF-1α expression during carcinogenesis in *H. pylori* infection and showed that HIF-1α was not expressed in the normal gastric mucosa, while expression increased in density and intensity with sequential progression from *H. pylori*-infected mucosa to gastric cancer [43]. Another study observed increased VEGF expression, HIF-1α target gene expression, and new microvessel formation in *H. pylori*-infected gastric mucosa [44]. Here, we studied the possible linkage between oncogenic CagA protein and HIF-1α expression. In *H. pylori*-infected gastric epithelial cells, CagA^+^ *H. pylori* increased not only HIF-1α protein levels, but also HIF-1α activity and the expression of HIF-1α-target genes, including *Vegf*, *Ldha,* and *Pdk1*, even under normoxic conditions *in vitro*. We strongly believe that *H. pylori* oncogenic CagA induces ROS production to stabilize HIF-1α and influences gastric cancer initiation and progression.

We also examined the relationship between SIRT3 activity and ROS-induced HIF-1α activation in *H. pylori* infection. Surprisingly, we observed lower levels of SIRT3 protein and activity in the mitochondria extracts of the CagA^+^ *H. pylori* infected cells. We attempted to identify a possible mechanism for the attenuation of SIRT3 in the mitochondria of CagA^+^ *H. pylori*-infected cells. Some studies have demonstrated that exposure of mammalian cells in culture to moderate oxidative stress significantly increases the degradation of intracellular proteins [45, 46]. The proteasome machinery appears to be upregulated in response to oxidative stress and to be responsible for the degradation of oxidized proteins in mammalian cells [45, 47]. We confirmed that SIRT3 proteins are restored by blocking proteasomal activation, suggesting that oxidative stress by *H. pylori* infection can degrade SIRT3 protein. This is, however, still unclear, and additional study is required to understand how the *H. pylori* oncogenic CagA induces the degradation of mitochondrial SIRT3. We also confirmed that the gain of function of SIRT3 can recover the metabolic change in *H. pylori* infected gastric cancer cells. We found that HIF-1α activity, ROS production, and HIF-1α target gene expression were relatively decreased in SIRT3 overexpressing cells with CagA^+^ *H. pylori* infection.

In conclusion, this study suggests that *H. pylori*-mediated gastric carcinogenesis could be attributed to hypoxic activation of HIF-1α activity via downregulation of mitochondrial SIRT3 in CagA^+^ *H. pylori*-infected gastric cancer. SIRT3 may be a valuable effector molecule for gastric cancer prevention, as well as a potential treatment strategy.

## MATERIALS AND METHODS

### Bacteria, cell culture, and infection with *H. pylori*

The *H. pylori* strains used were 60190 (cagPAI^+^, ATCC 49503), 8822 (cagPAI^-^), Δ*cagA* (an isogenic mutant of 60190 lacking *cagA*), and Δ*vacA* (an isogenic mutant of 60190 lacking *vacA*). *H. pylori* strains were cultured on agar plates containing 10% horse serum at 37°C in a microaerobic atmosphere, using the Campy Container System (BBL). Human gastric epithelial cells (AGS and MKN28) were cultured in RPMI-1640 medium (Gibco) supplemented with 10% fetal bovine serum (FBS) and 1% penicillin-streptomycin sulfate. All cultures were maintained in a 37°C incubator supplemented with 5% CO_2_. Gastric epithelial cells were seeded in tissue culture plates at 48 h before infection and allowed to grow to approximately 80% confluence. At 2 h prior to infection, the medium was replaced by fresh medium containing 0.5 % FBS. For infection, bacteria were harvested in phosphate-buffered saline (PBS) (pH 7.4) and added to the host cells at multiplicities of infection (MOIs) of 100 for varying times. Hypoxic conditions were generated by placing cells in a sealed hypoxic incubator (Thermo Scientific, MA, USA). The O_2_ level in this chamber was maintained at 1%, with the residual gas mixture containing 5% CO_2_ and 94% N_2_.

### Antibodies and chemicals

Anti-HIF-1α, anti-CagA, anti-PHD1, anti-PHD3, anti-Aldolase A, anti-Lamin B, and anti-β-actin were purchased from Santa Cruz Biotechnology, Inc. (Santa Cruz, CA, USA). An anti-HIF-1α antibody was also purchased from BD Biosciences (San Jose, CA, USA). Anti-SIRT3, anti-PHD2, anti-Cox IV, and anti-hydroxylated-HIF-1α were purchased from Cell Signaling Biotechnology (Danvers, MA, USA). The antioxidant inhibitors and chemicals were purchased from Sigma (St Louis, MO, USA).

### Immunoblotting

Whole cell extracts were prepared with RIPA buffer containing 50 mM Tris (pH 7.5), 1 mM EDTA, 150 mM NaCl, 0.1% SDS, 0.1% NaDeOC, 1% NP-40, and complete protease inhibitor (Roche Applied Science). Lysates were separated by SDS-PAGE and transferred to polyvinylidene difluoride membranes (Millipore, Billerica, MA, USA). Membranes were incubated with appropriate primary antibodies overnight at 4°C. Immunodetection was performed using an enhanced chemiluminescence reagent (Thermo Fisher Scientific), according to the manufacturer’s instructions.

### Plasmids

pcDAN3.1-SIRT3 Flag vector (No. 13814) was obtained from Addgene (Cambridge, MA, USA). To generate a lentiviral transfer vector with Flag-tagged SIRT3, pcDAN3.1-SIRT3 Flag vector was digested with restriction enzymes BamHI and XbaI and ligated into enzyme-digested pLVX-tight puro vector (Clontech, CA, USA). SIRT3 pLKO.1 shRNA vectors were purchased from Sigma (TRCN0000038892), and the control pLKO.1 scrambled vector was obtained from Addgene.

### Luciferase activity assay

HRE-luc plasmid (graciously provided by Prof. L. Guarente) was used to measure HIF-mediated transcriptional activity. Cells (3×10^5^) were plated into each well of a six-well plate and were transfected the next day using LipofectAMINE 2000 (Invitrogen) with 1 μg of HRE-luciferase plasmid, containing three copies of HRE from the *pgk-1* gene, and 0.05 μg of pRL-TK plasmid. At 24 h after transfection, cells were infected with *H. pylori* or incubated in a hypoxia chamber. Subsequently, cells were lysed, and luciferase activities were measured using a dual-luciferase reporter assay kit (Promega, Madison, WI, USA) according to the manufacturer’s protocol. Luciferase activity was measured using a luminometer (Berthold Technologies, Bad Wildbad, Germany). Values for firefly luciferase were normalized to Renilla (pRL-TK) vector and presented as fold induction, compared to control.

### Real-time PCR

Total RNA was isolated using TRIzol Reagent (Invitrogen) according to the manufacturer’s instructions. Complementary DNA was produced with SuperScript II reverse transcriptase (Invitrogen, Carlsbad, CA, USA) at 42°C for 1 h. The mixture was then boiled for 5 min to inactive reverse transcriptase and quickly chilled on ice. Synthesized cDNAs were analyzed by real-time PCR using SYBR Green master mix (Applied Biosystems, Foster City, CA, USA) and the ABI PRISM 7000 Quantitative PCR system (Applied Biosystems). Each reaction was performed in triplicate, and the amounts of the PCR products produced were normalized with respect to β-actin.

### Subcellular fractionation

Cytosolic and nuclear protein extraction: *H. pylori*-infected AGS cells were harvested and separated into cytoplasmic and nuclear fractions using CEB buffer (10 mM Tris-HCl, pH 8, 60 mM KCl, 1 mM EDTA, 1 mM dithiothreitol), containing 0.5% Nonidet P-40 and protease inhibitors. The cytoplasmic fraction was clarified by centrifugation at 1200 × *g* for 5 min, and the nuclear pellet was washed with CEB buffer to remove cytoplasmic contamination. The nuclear pellet was lysed with NEB buffer (20 mM Tris-HCl, pH 8, 0.4 M NaCl, 1.5 mM MgCl_2_, 1.5 mM EDTA, 1 mM dithiothreitol) containing 0.5% Nonidet P-40 and protease inhibitors. Nuclear proteins were analyzed by immunoblotting as described above.

Mitochondrial protein extraction: Mitochondrial and cytosolic fractions were extracted using a mitochondria isolation kit (Pierce Biotechnology, Inc., Rockford, IL, USA) following the manufacturer’s protocol. The cytoplasmic fraction was concentrated by acetone precipitation, and isolated mitochondria were lysed with RIPA buffer. Then equal amounts of protein were subjected to direct immunoblot analysis.

### ROS determination

Superoxide production was determined by measuring MitoSOX or DHE (dihydroethidium, Invitrogen) oxidation in cells, following the manufacturer’s instructions. Cells were cultured as described above, and the cells were washed once with PBS and incubated for 20 min at 37°C in RPMI-1640 containing 5 μM MitoSOX or 20 μM DHE before being trypsinized, resuspended, and measured by flow cytometry. For confocal immunofluorescence microscopy, *H. pylori*-infected cells were stained with MitoSOX (5 μM) for 20 min at 37°C, washed with PBS, and counterstained with mounting medium for fluorescence. Slides were sealed with coverslips and examined for immunofluorescence using a confocal laser-scanning microscope (LSM 700; Carl Zeiss, Thornwood, NY, USA).

### Immunofluorescence microscopy

*H. pylori*-infectedAGS cells were stained with Mitotracker for 30 min at 37°C, fixed in ice-cold methanol for 10 min, and washed with PBS. Nonspecific binding was blocked with 3% bovine serum albumin (BSA) in PBS for 30 min, followed by incubation with anti-CagA rabbit polyclonal antibody in 2% BSA in PBS at 4°C overnight. After washing with PBS, CagA was visualized by treatment with fluorescein isothiocyanate-conjugated goat anti-rabbit polyclonal secondary antibody for 60 min at room temperature. Cells were washed with PBS, mounting medium for fluorescence was added, and the slides were sealed with coverslips before examination for immunofluorescence using a confocal laser-scanning microscope.

### SIRT3 enzymatic activity assay

SIRT3 enzymatic activity was assayed with the Fluorogenic SIRT3 assay kit (BPS Bioscience, CA, USA) following the manufacturer’s instructions, with slight modifications. Mitochondrial protein (1 μg) was incubated at 37°C for 45 min with specific substrates; 25 μl of developer was added; and after 45 min of incubation, SIRT3 activity was measured using a fluorimetric microplate reader at 360 nm/460 nm.

### Immunohistochemical staining

To quantify angiogenesis, microvessel density was determined by counting CD31-positive vessels. Tissues from nude mice were fixed overnight in formalin at 4°C and embedded in paraffin. Tissue sections were deparaffinized with xylene, hydrated in serial dilutions of alcohol, and immersed in 3% H_2_O_2_. Following antigen retrieval by heat mediation in a Tris/EDTA buffer (pH 9) solution, the tissue sections were incubated with protein-blocking agent (Immunotech, Marseille, France) to block nonspecific antibody binding for 30 min at room temperature and then incubated overnight at 4°C with primary antibody against anti-CD31 (Abcam, 1:200) in a humidified chamber. After washing with PBS three times, the sections were incubated with a biotinylated secondary antibody and streptavidin conjugated to horseradish peroxidase (Immunotech) for 60 min at room temperature, followed by a PBS wash. The chromogen was developed for various times with liquid 3,3’-diaminobenzidine (Immunotech) followed by counterstaining with Meyer’s hematoxylin. Slides were examined under a light microscope.

### Xenografts

First, 5 × 10^6^ AGS cells were resuspended in 100 μl of serum-free RPMI-1640 medium with Matrigel and then injected subcutaneously into either flank of six-week-old BALB/c nude mice (OrientBio, Kapyoung, Korea). For the doxycycline-treated tumor group, AGS cells expressing SIRT3 were first treated with doxycycline (2 μg/ml) for 2 days to induce SIRT3 expression. 5 × 10^6^ of these cells in 100 μl serum-free RPMI-1640 medium with Matrigel were injected into the nude mice. After injection, mice were given doxycycline (1 mg/ml) with drinking water for three months. The bottles of water were changed weekly. The tumors were measured weekly by calipers, and their volumes were calculated as V = L × W^2^ × 0.5 (length L and width W). At the end of the experiment, mice were sacrificed and tumors were weighed. Tissues were removed for RT-PCR, immunoblotting, and IHC.

### Statistical analysis

Statistical analyses were performed using SPSS 18.0 software. All values are expressed as the mean ± standard deviation. Comparisons between two groups were analyzed using t-tests, and one-way ANOVA with a post hoc Bonferroni test was used for multiple comparisons. *P*-values <0.05 were considered statistically significant.

## SUPPLEMENTARY MATERIALS FIGURE


