# Supplementary material for: Regulation of SIRT3 signal related metabolic reprogramming in gastric cancer by Helicobacter pylori oncoprotein CagA
Source: Oncotarget. 2017 Jun 27;8(45):78365–78. doi: 10.18632/oncotarget.18695 (PMC5667968; doi:10.18632/oncotarget.18695)
Supplement: Supplementary file 1 [file oncotarget-08-78365-s001.pdf]

Regulation of SIRT3 signal related metabolic reprogramming in gastric cancer by *Helicobacter pylori* oncoprotein CagA

SUPPLEMENTARY MATERIALS

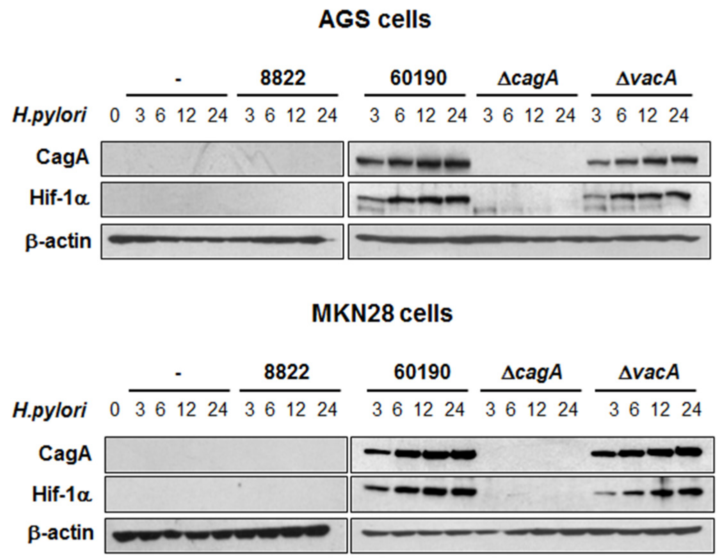

Supplementary Figure 1: HIF-1α protein expression was increased in *H. pylori* CagA dependent manner. Lysates from AGS and MKN28 cells infected with *H. pylori* strains at 0, 3, 6, 12, and 24 h were immunoblotted with anti-CagA, anti-HIF-1α, and anti-β-actin.
